# Dysregulation of antiviral helicase pathways in systemic lupus erythematosus

**DOI:** 10.3389/fgene.2014.00418

**Published:** 2014-11-25

**Authors:** Luciana Oliveira, Nailú A. Sinicato, Mariana Postal, Simone Appenzeller, Timothy B. Niewold

**Affiliations:** ^1^Rheumatology Unit, Department of Medicine, Faculty of Medical Science, State University of CampinasCampinas, Brazil; ^2^Mayo Clinic, Division of Rheumatology, Department of ImmunologyRochester, MN, USA

**Keywords:** antiviral helicase, systemic lupus erythematosus, interferon

## Abstract

In the autoimmune disease systemic lupus erythematosus (SLE), our normal antiviral defenses are inappropriately activated, resulting in over-activity of the type I interferon (IFN) pathway. This increased activity of the type I IFN pathway is an important primary pathogenic factor in the disease. Emerging evidence has implicated the antiviral helicases in this process. The antiviral helicases normally function as nucleic acid receptors in viral immunity. Genetic variations in antiviral helicase genes have been associated with SLE, supporting the idea that helicase pathways are involved in the primary pathogenesis of SLE. Studies have documented functional consequences of these genetic variations within the type I IFN pathway in human cell lines and SLE patients. In this review, we summarize the function of helicases in the anti-viral immune response, and how this response is dysregulated in SLE patients. In particular, we will focus on known functional genetic polymorphisms in the IFIH1 (MDA5) and mitochondrial antiviral signaling protein genes which have been implicated in human SLE. These data provide fascinating evidence for dysregulation of helicase-mediated innate immunity in SLE, and may support novel therapeutic strategies in the disease.

## INTRODUCTION

Systemic lupus erythematosus (SLE) is a chronic autoimmune disease characterized by multisystem inflammation commonly including the skin, kidneys, and joints, and other systems. While the pathogenesis of SLE is not completely understood, it seems likely that both genetic and environmental factors contribute to the disease. A number of genetic factors have been associated with SLE in recent years (Harley et al., 2008; Ghodke-Puranik and Niewold, 2013), providing a window into human disease pathogenesis. Interestingly, many of these genetic variations associated with risk of SLE have function within the type I interferon (IFN) pathway (Ghodke-Puranik and Niewold, 2013). Type I IFN is a classical anti-viral molecule which causes activation of antigen presenting cells within the innate immune system and increased expression of MHC and co-stimulatory molecules (Pestka et al., 2004).

Many lines of evidence support the idea that type I IFN plays a primary role in SLE pathogenesis (Niewold, 2011). Circulating type I IFN levels are elevated in many SLE patients (Weckerle et al., 2011), and this elevation is also observed in unaffected members of SLE families, suggesting that high IFN levels are a heritable risk factor for SLE (Niewold et al., 2007). Familial aggregation has been observed with other cytokines in SLE, such as tumor necrosis factor alpha and IL-10 (Grondal et al., 1999; Mangale et al., 2013), but in these cases unrelated family members such as spouses shared the trait as well, suggesting a contribution from environmental factors. Subsequent study of SLE-associated genetic factors has confirmed that SLE-risk genes contribute to the high IFN levels observed in SLE (Kariuki et al., 2008; Kariuki and Niewold, 2010; Agik et al., 2012; Niewold et al., 2012), and it seems that the high IFN trait is significantly polygenic (Harley et al., 2010; Kariuki et al., 2010; Koldobskaya et al., 2012; Jensen et al., 2013). The genetic data all support the concept that common gain-of-function variations in the type I IFN pathway are associated with SLE pathogenesis. Additionally, rare variants in the TREX1 gene have been described that are strongly associated with a SLE and Aicardi-Goutieres syndrome, a rare condition characterized by alterations in type I IFN and neurologic symptoms (Lee-Kirsch et al., 2007; Namjou et al., 2011). Recombinant human type I IFN has been administered as a therapeutic to treat some malignancies and chronic viral infection, and in some cases *de novo* SLE has developed (Ronnblom et al., 1990), which typically resolves when the type I IFN is stopped (Niewold and Swedler, 2005). These data taken together support the idea that type I IFN is a primary pathogenic factor in human SLE. While there are significant differences in SLE incidence between men and women (9:1 female), type I IFN pathway activation seems to be equal between men and women with SLE (Niewold et al., 2008a; Weckerle and Niewold, 2011). There is an increased incidence of SLE in African-Americans as compared to European-American populations (4:1), and in this case is seems that there are some differences in the way the pathway is activated, but high IFN is clearly seen in both populations (Ko et al., 2012, 2013). Thus type I IFN is a common pathway to SLE susceptibility, and it follows that molecules operating upstream of type I IFN production would play a role in disease. A large body of work has supports the relevance of the endosomal Toll-like receptors in SLE pathogenesis (Lovgren et al., 2004; Salloum and Niewold, 2011). In this review, we will focus on emerging data which implicates RNA helicases in type I IFN pathway dysregulation in human SLE. These data may also be relevant to other autoimmune diseases, as a number of conditions have been associated with increased type I IFN, including dermatomyositis, Sjogren’s syndrome, neuromyelitis optica, and others (Niewold et al., 2008b, 2011; Sweiss et al., 2011; Feng et al., 2012; Mavragani et al., 2013). In particular, we will focus on known functional genetic polymorphisms in the IFIH1 (MDA5) and mitochondrial antiviral signaling protein (MAVS) genes which function in helicase pathways, and have been implicated in human SLE.

## PATTERN RECOGNITION RECEPTORS INVOLVED IN ANTI-VIRAL RESPONSES AND SLE

**FIGURE 1:**
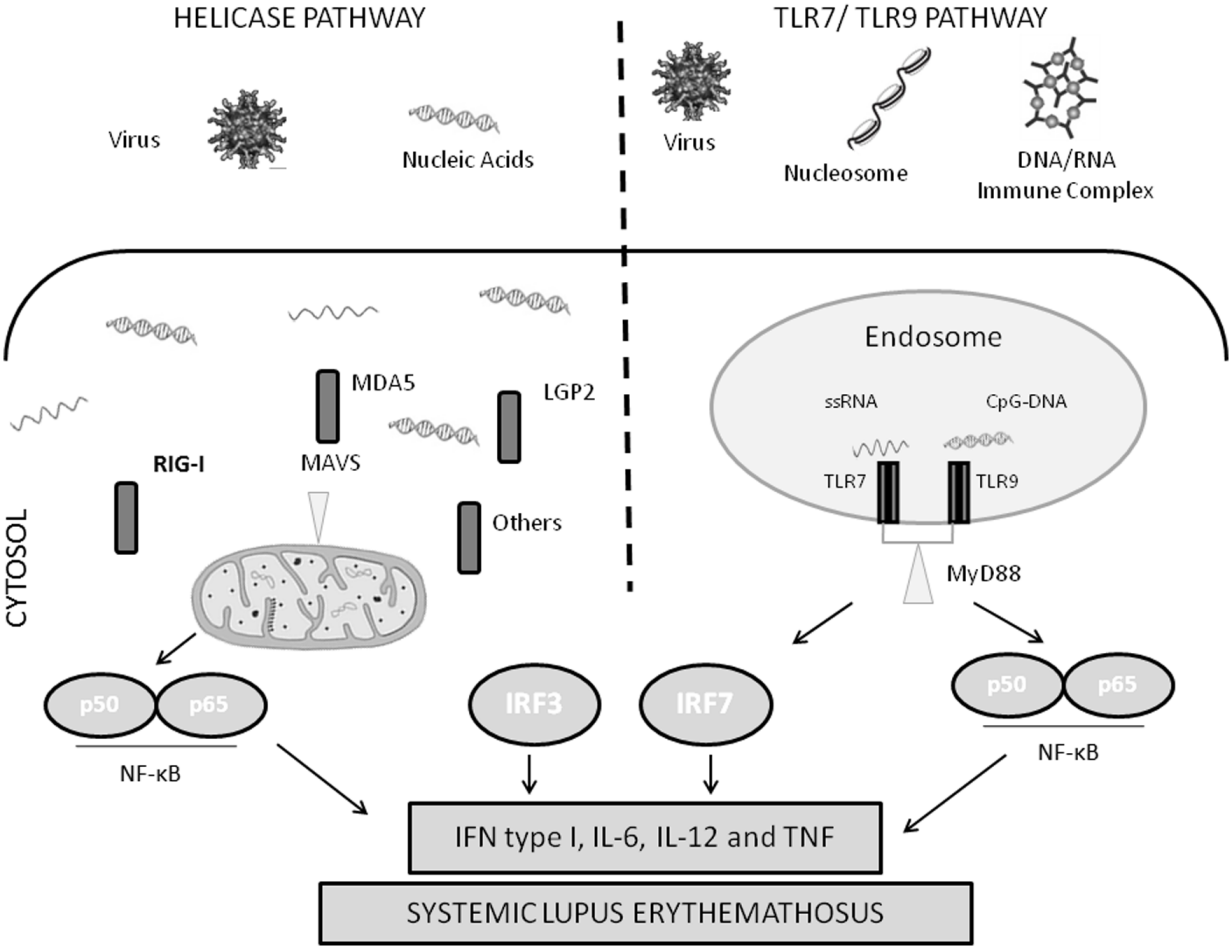
**Diagram of nucleic acid sensing antiviral immune response pathways.** IFN, type I interferon; IL, interleukin; IRF, interferon regulatory factor; LGP2, laboratory of genetics and physiology 2; MAVS, mitochondrial antiviral signaling protein; MDA5, melanoma differentiation-associated gene 5; MyD88, myeloid differentiation primary response gene 88; NF-κB, nuclear factor-κB; RIG-I, retinoic acid-inducible gene I; TLR, Toll-like receptor; TNF, tumor necrosis factor; TRIF, Toll/IL-1R-domain-containing adaptor inducing interferon β.

Several families of receptors that recognize pathogen-associated molecular patterns (PAMPs) have been described, such as the Toll-like receptors (TLRs) and retinoic acid-inducible gene I (RIG-I)-like receptor (RLRs). TLRs are transmembrane receptors expressed in specific immune cells, such as dendritic cells and macrophages. TLR7, 8, and 9 are expressed in the endosomal membrane, and can recognize viral nucleic acid. In anti-viral immunity, viral immune complexes are taken up via Fc receptors, and then delivered to the endosome resulting in TLR activation. RLRs, on the other hand, are cytosolic proteins that can recognize viral nucleic acid in the cytosol. Activation of either TLRs or RLRs results in IFN production and an anti-viral response (**Figure 1**).

The endosomal TLRs have been implicated in SLE pathogenesis in a number of previous studies. Genetic variations in TLR7 are associated with SLE susceptibility in humans (Deng et al., 2013). Mice which carry a duplication of the endosomal TLR region of the X-chromosome have enhanced susceptibility to an SLE-like disease (Subramanian et al., 2006). Interestingly, the autoantibodies which are characteristically produced in SLE target components of the cell nucleus, for example antibodies against double-stranded DNA and nuclear RNA-binding proteins. These SLE-associated autoantibodies form immune complexes that contain RNA and DNA, and the immune complexes can result in activation of the TLR system with subsequent type I IFN production (Lovgren et al., 2004, 2006). Thus, the anti-nuclear immune response that characterizes SLE produces immune complexes that are viral mimics, subverting normal viral immunity. These autoantibodies are frequently high titer and continuously present in SLE, and the antigens are ubiquitous, resulting in consistent inappropriate activation of the type I IFN pathway in SLE (Salloum and Niewold, 2011). Supporting this idea, one of the accepted and effective treatments for SLE, hydroxychloroquine, seems to interrupt endosomal TLR signaling (Kuznik et al., 2011; Sacre et al., 2012).

## RNA HELICASES

Ribonucleic acid helicases are involved in almost all cellular processes involving RNA (Steimer and Klostermeier, 2012). These enzymes use ATP to bind or remodel RNA and RNA–protein complexes (Linder and Jankowsky, 2011). Based on their shared conserved motifs and three dimensional core structures, RNA helicases are grouped into families and superfamilies (Linder and Jankowsky, 2011; Steimer and Klostermeier, 2012). The majority of RNA helicases belong to superfamily 2 (SF2; Pyle, 2008; Steimer and Klostermeier, 2012). DEAD box proteins are the largest family of helicases in SF2, and in humans these helicases have essential physiological roles in cellular RNA metabolism (Linder and Jankowsky, 2011). The DEAD box helicases work by destabilizing short RNA duplexes close to the binding site of the helicase. In contrast, the DExH group of helicases work in a progressive fashion, unwinding longer stretches of RNA (Pyle, 2008). DEAD box helicases frequently play a role in viral immunity by acting as sensors cytosolic viral nucleic acids. Besides the RLR DEAD box helicases which include RIG-I, MDA5, and LGP2, other DEAD box helicases likely perform this role as well, including DDX1, DDX3, DDX36, DDX41, DDX60, and others (Fullam and Schroder, 2013). In addition to sensing nucleic acid, some of these functions may be helicases further downstream in the pattern recognition receptor signaling pathways, potentially playing roles in transcriptional regulation (Fullam and Schroder, 2013). Interestingly, it appears that some RNA helicases are important for viral replication, suggesting that viruses have adopted this cellular mechanism to their own advantage in some cases (Fullam and Schroder, 2013).

Retinoic acid-inducible gene-I and MDA5 are proteins encoded by the DDX58 and IFIH1 genes, respectively. These RLRs are induced by type I IFN, and each recognize specific types of viruses (Yoneyama and Fujita, 2008). RIG-I and MDA5 recognize distinct viral RNA structures containing 5′ triphosphate in single and double-stranded RNA (Shrivastav and Niewold, 2013). These two RLRs demonstrate some specificity in the types of nucleic acids they recognize: while MDA5 senses picornavirus-derived nucleic acid, RIG-I senses other viral nucleic acids, such as those derived from influenza A (Yoneyama and Fujita, 2008). This differential recognition is based on the distinct RNA patterns generated by different viruses (Yoneyama and Fujita, 2008).

Activation of RIG-I and MDA5 by nucleic acid leads to binding of the mitochondrial adaptor IFN β promoter stimulater 1 (IPS-1) also known as MAVS (Reikine et al., 2014). As the name suggests, MAVS is found in the mitochondrial membrane, and is critical to signal transduction via MDA5 and RIG-I. MAVS forms large multimeric polymers on the mitochondrial membrane in combination with RIG-I and MDA5 bound to target nucleic acids, forming an active signaling complex (Reikine et al., 2014). This leads to activation of NF-κB, IRF3, and IRF7 (Reikine et al., 2014). These transcription factors are involved in IFN and interferon-stimulated gene expression, and the production of type-1 IFN and pro-inflammatory cytokines (Shrivastav and Niewold, 2013; **Figure 1**).

## MDA5/IFIH1

IFIH1 is the gene that encodes MDA5, and a common coding-change polymorphism in the IFIH1 gene has been associated with risk of SLE and other autoimmune diseases in humans (Smyth et al., 2006; Sutherland et al., 2007; Harley et al., 2008; Gateva et al., 2009; Strange et al., 2010; Molineros et al., 2013). This A946T polymorphism in IFIH1 was identified in case-control genetic studies of SLE (Harley et al., 2008; Gateva et al., 2009), and interestingly this polymorphism was the major finding in a recent admixture-mapping genetic screen to identify genes associated with SLE in African-Americans (Molineros et al., 2013), supporting relevance of this polymorphism across multiple ancestral backgrounds. This polymorphism appears to be gain-of-function in nature, being associated with increased IFIH1 mRNA expression (Downes et al., 2010), increased sensitivity to type I IFN and increased IFN-induced gene expression in circulating blood cells from SLE patients (Robinson et al., 2011), and modulation of inflammation- and apoptosis-related gene expression (Molineros et al., 2013). These studies support the general idea that over-activity of the anti-viral helicases would result in greater type I IFN signaling and risk of SLE. Rare loss-of-function variations in IFIH1 have been discovered in the IFIH1 gene, and interestingly these loss-of-function variants appear to be protective against autoimmune disease (Nejentsev et al., 2009), further supporting the idea that increased function of IFIH1/MDA5 is associated with risk of autoimmune disease.

Studies in mice have also supported this hypothesis. A recent study demonstrated that a single coding-change mutation in IFIH1 (Gly821Ser) generated by *N*-ethyl-*N*-nitrosourea (ENU) resulted in constitutive activation of MDA5 (Funabiki et al., 2014). Mice with this mutation developed a systemic autoimmune disease similar to lupus, with nephritis characterized by lymphocyte infiltration as well as deposition of immunoglobulin and complement, systemic inflammation in the heart and lung, and increased tumor necrosis factor alpha, IL-6 and type I IFN (Funabiki et al., 2014). This gain-of-function mutation in the mouse line has not been observed in humans, but it supports the general concept that gain of function in IFIH1 is associated with autoimmunity.

## MAVS

Genetic studies in human SLE have also identified a functional coding-change polymorphism in MAVS, a key adapter of both the RIG-I and MDA5 helicases. The C97F polymorphism in MAVS substantially reduced the expression of type I IFN and other proinflammatory mediators in human cell lines (Pothlichet et al., 2011). Interestingly, this variation was almost exclusively found in the African-American population, with a frequency of 10.2% in controls (Pothlichet et al., 2011). In African–American patients with SLE, the C79F allele was associated with low type I IFN and was more than twice as common (22.4% frequency) in SLE patients who lacked autoantibodies to RNA-binding proteins. This study demonstrated that a coding-change genetic variation in the gene encoding MAVS has a functional impact on the antiviral IFN pathway in humans, and is associated with a serologic subgroup of SLE patients (Pothlichet et al., 2011). These studies in both MAVS and IFIH1 demonstrate the importance of variations in these genes upon immune function and autoimmune disease risk in human populations.

## CONCLUSION

Dysregulation of anti-viral helicase immune responses represent a primary pathogenic factor in human SLE. This is demonstrated by the presence of coding-change polymorphisms in both the IFIH1 and MAVS genes which modulate function of the type I IFN pathway and risk of SLE in humans. While immune complexes formed by SLE autoantibodies and nuclear material seem to be the likely trigger for endosomal TLR stimulation in SLE, the exact triggers of the cytosolic anti-viral helicases in human SLE are somewhat less clear. Viruses may stimulate some of the early events in SLE patients, as strong epidemiological data implicates Epstein–Barr virus infection in the initial pathogenesis of SLE (James et al., 1997; Poole et al., 2006). It is possible that this represents a gene – environment interaction in human SLE – a hypersensitive or overactive anti-viral helicase system coupled with a viral trigger, such as Epstein–Barr virus infection, which then results in an exaggerated type I IFN response and subsequent misdirection of the adaptive immune response against self-antigens. It is also possible that viral-like elements, such as LINE elements, may also play a role in the chronic stimulation of these cytosolic nucleic acid receptors (Crow, 2010). Both Epstein-Barr virus and LINE-1 RNAs could potentially be seen as “foreign” by the cytosolic helicases. The fact that both the TLR and cytosolic pathways of viral recognition are involved in human SLE and the convergence of these pathways upon type I IFN and anti-viral responses is striking, suggesting that over-active anti-viral immunity represents a major common pathogenic pathway in human SLE. A number of therapeutics have been developed to target type I IFN in SLE, including monoclonal antibodies against IFN-α (Merrill et al., 2011; Kalunian et al., 2012; McBride et al., 2012), as well as a vaccination strategy aimed at inducing antibodies against IFN-α (Lauwerys et al., 2013). These studies are in early stages, phase I to phase II, and thus far the data generally support relative safety and proof-of-mechanism. It is too early to make conclusions about efficacy, but some of the larger phase II studies have reported potential subset effects within the overall SLE patient groups (Kalunian et al., 2012). This would suggest that anti-IFN therapies may not work for every patient, but the genetics data we discuss in this review may also suggest this outcome. It seems that the IFN pathway is impacted by a number of genetic factors, and these factors will not be shared by all patients. Therapeutics targeting the RNA helicases could be potentially interesting in SLE, and further understanding of the specific dysregulation of the helicase pathways in human SLE such as the work summarized in this review could help to determine optimal points of intervention in the pathway and in which group of patients.

## Conflict of Interest Statement

The authors declare that the research was conducted in the absence of any commercial or financial relationships that could be construed as a potential conflict of interest.

## References

[B1] Agik S. Franek B. S. Kumar A. A. Kumabe M. Utset T. O. Mikolaitis R. A. (2012). The autoimmune disease risk allele of UBE2L3 in African American patients with systemic lupus erythematosus: a recessive effect upon subphenotypes. *J. Rheumatol.* 39 73–78 10.3899/jrheum.110590 22045845 PMC3304461

[B2] Crow M. K. (2010). Long interspersed nuclear elements (LINE-1): potential triggers of systemic autoimmune disease. *Autoimmunity* 43 7–16 10.3109/08916930903374865 19961365

[B3] Deng Y. Zhao J. Sakurai D. Kaufman K. M. Edberg J. C. Kimberly R. P. (2013). MicroRNA-3148 modulates allelic expression of toll-like receptor 7 variant associated with systemic lupus erythematosus. *PLoS Genet.* 9:e1003336 10.1371/journal.pgen.1003336 PMC3585142 23468661

[B4] Downes K. Pekalski M. Angus K. L. Hardy M. Nutland S. Smyth D. J. (2010). Reduced expression of IFIH1 is protective for type 1 diabetes. *PLoS ONE* 55:e12646 10.1371/journal.pone.0012646 PMC2936573 20844740

[B5] Feng X. Reder N. P. Yanamandala M. Hill A. Franek B. S. Niewold T. B. (2012). Type I interferon signature is high in lupus and neuromyelitis optica but low in multiple sclerosis. *J. Neurol. Sci.* 313 48–53 10.1016/j.jns.2011.09.032 22036215 PMC3910514

[B6] Fullam A. Schroder M. (2013). DExD/H-box RNA helicases as mediators of anti-viral innate immunity and essential host factors for viral replication. *Biochim. Biophys. Acta* 1829 854–865 10.1016/j.bbagrm.2013.03.012 23567047 PMC7157912

[B7] Funabiki M. Kato H. Miyachi Y. Toki H. Motegi H. Inoue M. (2014). Autoimmune disorders associated with gain of function of the intracellular sensor MDA5. *Immunity* 40 199–212 10.1016/j.immuni.2013.12.014 24530055

[B8] Gateva V. Sandling J. K. Hom G. Taylor K. E. Chung S. A. Sun X. (2009). A large-scale replication study identifies TNIP1, PRDM1, JAZF1, UHRF1BP1 and IL10 as risk loci for systemic lupus erythematosus. *Nat. Genet.* 41 1228–1233 10.1038/ng.468 19838195 PMC2925843

[B9] Ghodke-Puranik Y. Niewold T. B. (2013). Genetics of the type I interferon pathway in systemic lupus erythematosus. *Int. J. Clin. Rheumtol.* 8 657–669 10.2217/ijr.13.58 PMC3885171 24416080

[B10] Grondal G. Kristjansdottir H. Gunnlaugsdottir B. Arnason A. Lundberg I. Klareskog L. (1999). Increased number of interleukin-10-producing cells in systemic lupus erythematosus patients and their first-degree relatives and spouses in Icelandic multicase families. *Arthritis Rheum.* 42 1649–1654 10.1002/1529-0131(199908)42:8<1649::AID-ANR13>3.0.CO;2-D 10446864

[B11] Harley J. B. Alarcon-Riquelme M. E. Criswell L. A. Jacob C. O. Kimberly R. P. Moser K. L. (2008). Genome-wide association scan in women with systemic lupus erythematosus identifies susceptibility variants in ITGAM, PXK, KIAA1542 and other loci. *Nat. Genet.* 40 204–210 10.1038/ng.81 18204446 PMC3712260

[B12] Harley T. W. I. Niewold T. B. Stormont R. M. Kaufman K. M. Glenn S. B. Franek B. S. (2010). The role of genetic variation near interferon-kappa in systemic lupus erythematosus. *J. Biomed. Biotechnol.* 2010:706825 10.1155/2010/706825 PMC2914299 20706608

[B13] James J. A. Kaufman K. M. Farris A. D. Taylor-Albert E. Lehman T. J. Harley J. B. (1997). An increased prevalence of Epstein-Barr virus infection in young patients suggests a possible etiology for systemic lupus erythematosus. *J. Clin. Invest.* 100 3019–3026 10.1172/JCI119856 9399948 PMC508514

[B14] Jensen M. A. Patterson K. C. Kumar A. A. Kumabe M. Franek B. S. Niewold T. B. (2013). Functional genetic polymorphisms in ILT3 are associated with decreased surface expression on dendritic cells and increased serum cytokines in lupus patients. *Ann. Rheum. Dis.* 72 596–601 10.1136/annrheumdis-2012-202024 22904259 PMC3910490

[B15] Kalunian K. Merrill J. T. Maciuca R. Ouyang W. McBride J. M. Townsend M. J. (2012). Efficacy and safety of rontalizumab (Anti-Interferon Alpha) in SLE subjects with restricted immunosuppressant use: results of a randomized, double-blind, placebo-controlled phase 2 study. *Arthritis Rheum.* 64:2622 10.1002/art.40353

[B16] Kariuki S. N. Crow M. K. Niewold T. B. (2008). The PTPN22 C1858T polymorphism is associated with skewing of cytokine profiles toward high interferon-alpha activity and low tumor necrosis factor alpha levels in patients with lupus. *Arthritis Rheum.* 58 2818–2823 10.1002/art.23728 18759295 PMC2621106

[B17] Kariuki S. N. Franek B. S. Kumar A. A. Arrington J. Mikolaitis R. A. Utset T. O. (2010). Trait-stratified genome-wide association study identifies novel and diverse genetic associations with serologic and cytokine phenotypes in systemic lupus erythematosus. *Arthritis Res. Ther.* 12:R151 10.1186/ar3101 PMC2945049 20659327

[B18] Kariuki S. N. Niewold T. B. (2010). Genetic regulation of serum cytokines in systemic lupus erythematosus. *Transl. Res.* 155 109–117 10.1016/j.trsl.2009.08.012 20171594 PMC2827336

[B19] Ko K. Franek B. S. Marion M. Kaufman K. M. Langefeld C. D. Harley J. B. (2012). Genetic ancestry, serum interferon-alpha activity, and autoantibodies in systemic lupus erythematosus. *J. Rheumatol.* 39 1238–1240 10.3899/jrheum.111467 22505704 PMC3381952

[B20] Ko K. Koldobskaya Y. Rosenzweig E. Niewold T. B. (2013). Activation of the interferon pathway is dependent upon autoantibodies in African-American SLE patients, but Not in European-American SLE patients. *Front. Immunol.* 4:309 10.3389/fimmu.2013.00309 PMC3787392 24101921

[B21] Koldobskaya Y. Ko K. Kumar A. A. Agik S. Arrington J. Kariuki S. N. (2012). Gene-expression-guided selection of candidate loci and molecular phenotype analyses enhance genetic discovery in systemic lupus erythematosus. *Clin. Dev. Immunol.* 2012:682018 10.1155/2012/682018 PMC3439981 22988468

[B22] Kuznik A. Bencina M. Svajger U. Jeras M. Rozman B. Jerala R. (2011). Mechanism of endosomal TLR inhibition by antimalarial drugs and imidazoquinolines. *J. Immunol.* 186 4794–4804 10.4049/jimmunol.1000702 21398612

[B23] Lauwerys B. R. Hachulla E. Spertini F. Lazaro E. Jorgensen C. Mariette X. (2013). Down-regulation of interferon signature in systemic lupus erythematosus patients by active immunization with interferon alpha-kinoid. *Arthritis Rheum.* 65 447–456 10.1002/art.37785 23203821

[B24] Lee-Kirsch M. A. Gong M. Chowdhury D. Senenko L. Engel K. Lee Y. A. (2007). Mutations in the gene encoding the 3′-5′ DNA exonuclease TREX1 are associated with systemic lupus erythematosus. *Nat. Genet.* 39 1065–1067 10.1038/ng2091 17660818

[B25] Linder P. Jankowsky E. (2011). From unwinding to clamping – the DEAD box RNA helicase family. *Nat. Rev. Mol. Cell Biol.* 12 505–516 10.1038/nrm3154 21779027

[B26] Lovgren T. Eloranta M. L. Bave U. Alm G. V. Ronnblom L. (2004). Induction of interferon-alpha production in plasmacytoid dendritic cells by immune complexes containing nucleic acid released by necrotic or late apoptotic cells and lupus IgG. *Arthritis Rheum.* 50 1861–1872 10.1002/art.20254 15188363

[B27] Lovgren T. Eloranta M. L. Kastner B. Wahren-Herlenius M. Alm G. V. Ronnblom L. (2006). Induction of interferon-alpha by immune complexes or liposomes containing systemic lupus erythematosus autoantigen- and Sjogren’s syndrome autoantigen-associated RNA. *Arthritis Rheum.* 54 1917–1927 10.1002/art.21893 16729300

[B28] Mangale D. Kariuki S. N. Chrabot B. S. Kumabe M. Kelly J. A. Harley J. B. (2013). Familial aggregation of high tumor necrosis factor alpha levels in systemic lupus erythematosus. *Clin. Dev. Immunol.* 2013:267430 10.1155/2013/267430 PMC3800640 24187561

[B29] Mavragani C. P. Niewold T. B. Chatzigeorgiou A. Danielides S. Thomas D. Kirou K. A. (2013). Increased serum type I interferon activity in organ-specific autoimmune disorders: clinical, imaging, and serological associations. *Front. Immunol.* 4:238 10.3389/fimmu.2013.00238 PMC3746787 23966997

[B30] McBride J. M. Jiang J. Abbas A. R. Morimoto A. Li J. Maciuca R. (2012). Safety and pharmacodynamics of rontalizumab in patients with systemic lupus erythematosus: results of a phase I, placebo-controlled, double-blind, dose-escalation study. *Arthritis Rheum.* 64 3666–3676 10.1002/art.34632 22833362

[B31] Merrill J. T. Wallace D. J. Petri M. Kirou K. A. Yao Y. White W. I. (2011). Lupus interferon skin activity study, safety profile and clinical activity of sifalimumab, a fully human anti-interferon alpha monoclonal antibody, in systemic lupus erythematosus: a phase I, multicentre, double-blind randomised study. *Ann. Rheum. Dis.* 70 1905–1913 10.1136/ard.2010.144485 21798883

[B32] Molineros J. E. Maiti A. K. Sun C. Looger L. L. Han S. Kim-Howard X. (2013). Admixture mapping in lupus identifies multiple functional variants within IFIH1 associated with apoptosis, inflammation, and autoantibody production. *PLoS Genet.* 9:e1003222 10.1371/journal.pgen.1003222 PMC3575474 23441136

[B33] Namjou B. Kothari P. H. Kelly J. A. Glenn S. B. Ojwang J. O. Adler A. (2011). Evaluation of the TREX1 gene in a large multi-ancestral lupus cohort. *Genes Immun.* 12 270–279 10.1038/gene.2010.73 21270825 PMC3107387

[B34] Nejentsev S. Walker N. Riches D. Egholm M. Todd J. A. (2009). Rare variants of IFIH1, a gene implicated in antiviral responses, protect against type 1 diabetes. *Science* 324 387–389 10.1126/science.1167728 19264985 PMC2707798

[B35] Niewold T. B. (2011). Interferon alpha as a primary pathogenic factor in human lupus. *J. Interferon Cytokine Res.* 31 887–892 10.1089/jir.2011.0071 21923413 PMC3234490

[B36] Niewold T. B. Adler J. E. Glenn S. B. Lehman T. J. Harley J. B. Crow M. K. (2008a). Age- and sex-related patterns of serum interfeon-alpha activity in lupus families. *Arthritis Rheum.* 58 2113–2119 10.1002/art.23619 18576315 PMC2729701

[B37] Niewold T. B. Rivera T. L. Buyon J. P. Crow M. K. (2008b). Serum type I interferon activity is dependent on maternal diagnosis in anti-SSA/Ro-positive mothers of children with neonatal lupus. *Arthritis Rheum.* 58 541–546 10.1002/art.23191 18240214 PMC2755051

[B38] Niewold T. B. Hua J. Lehman T. J. Harley J. B. Crow M. K. (2007). High serum IFN-alpha activity is a heritable risk factor for systemic lupus erythematosus. *Genes Immun.* 8 492–502 10.1038/sj.gene.6364408 17581626 PMC2702174

[B39] Niewold T. B. Kelly J. A. Kariuki S. N. Franek B. S. Kumar A. A. Kaufman K. M. (2012). IRF5 haplotypes demonstrate diverse serological associations which predict serum interferon alpha activity and explain the majority of the genetic association with systemic lupus erythematosus. *Ann. Rheum. Dis.* 71 463–468 10.1136/annrheumdis-2011-200463 22088620 PMC3307526

[B40] Niewold T. B. Swedler W. I. (2005). Systemic lupus erythematosus arising during interferon-alpha therapy for cryoglobulinemic vasculitis associated with hepatitis C. *Clin. Rheumatol.* 24 178–181 10.1007/s10067-004-1024-2 15565395

[B41] Niewold T. B. Wu S. C. Smith M. Morgan G. A. Pachman L. M. (2011). Familial aggregation of autoimmune disease in juvenile dermatomyositis. *Pediatrics* 127 e1239–e1246 10.1542/peds.2010-3022 21502224 PMC3081190

[B42] Pestka S. Krause C. D. Walter M. R. (2004). Interferons, interferon-like cytokines, and their receptors. *Immunol. Rev.* 202 8–32 10.1111/j.0105-2896.2004.00204.x 15546383

[B43] Poole B. D. Scofield R. H. Harley J. B. James J. A. (2006). Epstein-Barr virus and molecular mimicry in systemic lupus erythematosus. *Autoimmunity* 39 63–70 10.1080/08916930500484849 16455583

[B44] Pothlichet J. Niewold T. B. Vitour D. Solhonne B. Crow M. K. Si-Tahar M. (2011). A loss-of-function variant of the antiviral molecule MAVS is associated with a subset of systemic lupus patients. *EMBO Mol. Med.* 3 142–152 10.1002/emmm.201000120 21268286 PMC3395111

[B45] Pyle A. M. (2008). Translocation and unwinding mechanisms of RNA and DNA helicases. *Annu. Rev. Biophys.* 37 317–336 10.1146/annurev.biophys.37.032807.125908 18573084

[B46] Reikine S. Nguyen J. B. Modis Y. (2014). Pattern recognition and signaling mechanisms of RIG-I and MDA5. *Front. Immunol.* 5:342 10.3389/fimmu.2014.00342 PMC4107945 25101084

[B47] Robinson T. Kariuki S. N. Franek B. S. Kumabe M. Kumar A. A. Badaracco M. (2011). Autoimmune disease risk variant of IFIH1 is associated with increased sensitivity to IFN-alpha and serologic autoimmunity in lupus patients. *J. Immunol.* 187 1298–1303 10.4049/jimmunol.1100857 21705624 PMC3304466

[B48] Ronnblom L. E. Alm G. V. Oberg K. E. (1990). Possible induction of systemic lupus erythematosus by interferon-alpha treatment in a patient with a malignant carcinoid tumour. *J. Intern. Med.* 227 207–210 10.1111/j.1365-2796.1990.tb00144.x 1690258

[B49] Sacre K. Criswell L. A. McCune J. M. (2012). Hydroxychloroquine is associated with impaired interferon-alpha and tumor necrosis factor-alpha production by plasmacytoid dendritic cells in systemic lupus . *Arthritis Res. Ther.* 14:R155 10.1186/ar3895 PMC3446541 22734582

[B50] Salloum R. Niewold T. B. (2011). Interferon regulatory factors in human lupus pathogenesis. *Transl. Res.* 157 326–331 10.1016/j.trsl.2011.01.006 21575916 PMC3096827

[B51] Shrivastav M. Niewold T. B. (2013). Nucleic acid sensors and type I interferon production in systemic lupus erythematosus. *Front. Immunol.* 4:319 10.3389/fimmu.2013.00319 PMC3791549 24109483

[B52] Smyth D. J. Cooper J. D. Bailey R. Field S. Burren O. Smink L. J. (2006). A genome-wide association study of nonsynonymous SNPs identifies a type 1 diabetes locus in the interferon-induced helicase (IFIH1) region. *Nat. Genet.* 38 617–619 10.1038/ng1800 16699517

[B53] Steimer L. Klostermeier D. (2012). RNA helicases in infection and disease. *RNA Biol.* 9 751–771 10.4161/rna.20090 22699555

[B54] Strange A. Capon F. Spencer C. C. Knight J. Weale M. E. Allen M. H. (2010). A genome-wide association study identifies new psoriasis susceptibility loci and an interaction between HLA-C and ERAP1. *Nat. Genet.* 42 985–990 10.1038/ng.694 20953190 PMC3749730

[B55] Subramanian S. Tus K. Li Q. Z. Wang A. Tian X. H. Zhou J. (2006). A Tlr7 translocation accelerates systemic autoimmunity in murine lupus. *Proc. Natl. Acad. Sci. U.S.A.* 103 9970–9975 10.1073/pnas.0603912103 16777955 PMC1502563

[B56] Sutherland A. Davies J. Owen C. J. Vaikkakara S. Walker C. Cheetham T. D. (2007). Genomic polymorphism at the interferon-induced helicase (IFIH1) locus contributes to Graves’ disease susceptibility. *J. Clin. Endocrinol. Metab.* 92 3338–3341 10.1210/jc.2007-0173 17535987 PMC6952273

[B57] Sweiss N. J. Zhang W. Franek B. S. Kariuki S. N. Moller D. R. Patterson K. C. (2011). Linkage of type I interferon activity and TNF-alpha levels in serum with sarcoidosis manifestations and ancestry. *PLoS ONE* 6:e29126 10.1371/journal.pone.0029126.t001 PMC3237595 22195005

[B58] Weckerle C. E. Franek B. S. Kelly J. A. Kumabe M. Mikolaitis R. A. Green S. L. (2011). Network analysis of associations between serum interferon-alpha activity, autoantibodies, and clinical features in systemic lupus erythematosus. *Arthritis Rheum.* 63 1044–1053 10.1002/art.30187 21162028 PMC3068224

[B59] Weckerle C. E. Niewold T. B. (2011). The unexplained female predominance of systemic lupus erythematosus: clues from genetic and cytokine studies. *Clin. Rev. Allergy. Immunol.* 40 42–49 10.1007/s12016-009-8192-4 20063186 PMC2891868

[B60] Yoneyama M. Fujita T. (2008). Structural mechanism of RNA recognition by the RIG-I-like receptors. *Immunity* 29 178–181 10.1016/j.immuni.2008.07.009 18701081

